# *Polyporus squamosus* Lectin 1a (PSL1a) Exhibits Cytotoxicity in Mammalian Cells by Disruption of Focal Adhesions, Inhibition of Protein Synthesis and Induction of Apoptosis

**DOI:** 10.1371/journal.pone.0170716

**Published:** 2017-01-23

**Authors:** Dipankar Manna, Sascha Pust, Maria L. Torgersen, Gabriele Cordara, Markus Künzler, Ute Krengel, Kirsten Sandvig

**Affiliations:** 1 Department of Chemistry, University of Oslo, Oslo, Norway; 2 Department of Molecular Cell Biology, Institute for Cancer Research, Oslo University Hospital, Oslo, Norway; 3 Centre for Cancer Biomedicine, Faculty of Medicine, University of Oslo, Oslo, Norway; 4 Institute of Microbiology, ETH Zürich, Zürich, Switzerland; 5 Department of Biosciences, Faculty of Mathematics and Natural Sciences, University of Oslo, Oslo, Norway; Seoul National University College of Pharmacy, REPUBLIC OF KOREA

## Abstract

PSL1a is a lectin from the mushroom *Polyporus squamosus* that binds to sialylated glycans and glycoconjugates with high specificity and selectivity. In addition to its N-terminal carbohydrate-binding domain, PSL1a possesses a Ca^2+^-dependent proteolytic activity in the C-terminal domain. In the present study, we demonstrate that PSL1a has cytotoxic effects on mammalian cancer cells, and we show that the cytotoxicity is dependent on the cysteine protease activity. PSL1a treatment leads to cell rounding and detachment from the substratum, concomitant with disruption of vinculin complexes in focal adhesions. We also demonstrate that PSL1a inhibits protein synthesis and induces apoptosis in HeLa cells, in a time- and concentration-dependent manner.

## Introduction

Lectins are carbohydrate-binding proteins or glycoproteins that contain at least one carbohydrate-binding domain [1]. A few lectins contain, in addition to the carbohydrate-binding domain, a catalytic domain and are classified as chimerolectins. The carbohydrate-binding domain plays an important role in recognition and reversible binding to diverse glycotopes [2]. Several lectins contain multivalent sugar-binding domains giving them the ability to agglutinate cells and precipitate glycoconjugates [3, 4]. Lectins are widely distributed in nature and have been isolated from archaea to bacteria [5, 6], viruses [7], animals [8], plants and yeasts [9, 10]. These proteins play an important role in various biological processes, such as cell signaling, protein trafficking, and carcinogenesis [11, 12].

Mushrooms contain a wide variety of lectins, which play a significant role in host defense against insects and nematodes [13–15]. Several mushroom lectins have been reported to be antitumor proteins [16], and some of them are known as active proteases, such as the mushroom lectin *Marasmius oreades* agglutinin (MOA) [17, 18].

PSL1a is the closest homolog of MOA (38% sequence identity). It is a 286 amino acid homodimeric lectin from the mushroom *Polyporus squamosus*, which selectively binds to sialylated glycans and glycoconjugates [19]. The first crystal structure of PSL1a was reported in complex with the human-type influenza receptor Neu5Acα2-6Galβ1-4GlcNAc [20]. The non-catalytic N-terminal domain is involved in carbohydrate binding, whereas the C-terminal dimerization domain carries proteolytic activity [17].

Lectins that target sialic acid structures have long been studied in bacterial and viral adhesion because of their high selectivity [21]. In many cancer cells, malignancy is correlated with overexpression of sialic acids on the cell surface [22]. It has further been shown that secreted sialylated mucins can be detected in the bloodstream of cancer patients [23], and lectins are valuable diagnostic tools [24–26]. Thus, sialic acid specific lectins are useful tools for the detection and characterization of sialylated glycoconjugates displayed on the surface of cancer cells and tissues [27, 28].

In this study, we characterized for the first time the cytotoxic effect of the fungal lectin PSL1a on mammalian cancer cells. PSL1a treatment of cancer cells lead to cell rounding and detachment from the substratum in a time- and concentration-dependent manner. Microscopic studies show that exposure to PSL1a results in the disruption of cellular focal adhesion points, without a direct effect on the actin cytoskeleton. We further demonstrate that PSL1a leads to inhibition of protein synthesis and induction of apoptosis, but not autophagy.

## Materials and Methods

### Reagents and antibodies

Gels and buffers for Western blotting were purchased from Bio-Rad Laboratories Inc. (Hercules, CA, USA). BSA, lysozyme, concanamycin A, staurosporine, and E-64 inhibitor were purchased from Sigma-Aldrich (St. Louis, MO, USA). Dulbecco’s modified eagle medium (DMEM), penicillin, fetal bovine serum (FBS), and Earle’s balanced salt solution (EBSS) were purchased from Invitrogen Life Technologies, CA. QuikChange II kit from Stratagene, ArcticExpress (DE3) *Escherichia coli* cells from Agilent Technologies and 10000 MWCO PES membranes were purchased from Sartorius AG.

### Cell lines and cell culture

HeLa (ATCC: CCL-2), HEp-2 (ATCC: CCL-23), SKBR-3 (ATCC: HTB-30) and PC3 cells (ATCC: CRL-1435) were cultured in DMEM complemented with 10% (v/v) FBS, 2 mM L-glutamine, 50 U/ml penicillin and 50 mg/ml streptomycin. The non-cancer cell line hTERT RPE (ATCC: CRL-4000) was cultured in DMEM/F12 with 10% (v/v) FBS, and 0.01 mg/ml hygromycin B. Cells were seeded at 2×10^5^ cells/well in 6-well plates or at 5×10^4^ cells/well in 24-well plates 24 h prior to experiments and incubated at 37°C in a 5% CO_2_ incubator.

### Expression and purification of PSL1a

The PSL1a gene inserted in pET43.1a expression vector was a gift from Dr. Hiroaki Tateno (AIST, Japan). For expression of the proteolytically inactive PSL1a mutant (C208A), a mutation was introduced at the codon 208 by site-directed mutagenesis using QuikChange II kit (Stratagene) following the protocol provided by the manufacturer. The pET43.1a-PSL1a construct containing the cDNA for the wild-type (WT) or the C208A mutant were transformed in ArcticExpress (DE3) *Escherichia coli* cells (Agilent Technologies). Protein expression was induced with 0.1 mM IPTG, the bacteria were grown at 11°C for 24 h and were subsequently collected by centrifugation (5000 rcf, 15 min). The pellet was resuspended in a lysis buffer containing 50 mM Tris pH 8.0, 0.15 M NaCl, 2 mM EDTA, 1x concentrated c*O*mplete protease inhibitor cocktail EDTA free (Roche Diagnostics Ltd.), 1 μl/ml benzonase nuclease (Thermo Scientific) and 4 mg/ml hen egg white lysozyme (Sigma-Aldrich). After 2 h of incubation at room temperature, the insoluble fraction was discarded by centrifugation (20000 rcf, 45 min). The proteins were purified using a two-step chromatographic protocol. As the capture step, the clarified cell lysate was passed through a D-Gal-sepharose affinity resin (Thermo Scientific), followed by extensive washing with 20 mM imidazole pH 8.0 buffer and elution by using a 1.0 M D-Gal single step gradient. The fraction containing the eluted protein was concentrated using a 10000 MWCO PES membrane (Vivaspin, Sartorius AG) to a volume of approximately 500 μl and passed through Superdex 75 10/300 GL gel-filtration column (Tricorn, GE Healthcare Life Sciences) with a buffer containing 20 mM imidazole pH 8.0, 2 mM EDTA, 0.2 M D-Gal, 0.15 M NaCl and 2 mM DTT. The fractions containing the purified proteins were pooled, concentrated to 10–15 mg/ml using 10000 MWCO PES membrane concentrator and underwent three rounds of buffer exchange with 20 mM imidazole pH 8.0, 2 mM EDTA and 2 mM DTT. For live cell imaging, PSL1a was fluorescently labeled with Alexa Fluor 488 by using a Microscale Labeling Kit according to the manufacturer’s protocol (Invitrogen).

### Morphological characterizations of PSL1a treated cells

Cells were treated with 5 μg/ml of PSL1a in serum-free medium and incubated for 4 h at 37°C. RPE cells were also incubated with PSL1a (0.5 and 5 μg/ml) for 24 and 48 h. The cell morphology was monitored using a Nikon Eclipse TS100 microscope and the number of rounded cells was analyzed. The images were captured with a Nikon DS-Fi1 HD color camera.

### Confocal microscopy, 3D SIM and live cell imaging

Cells were cultured as described before and seeded on coverslips. The cells were washed in PBS and then fixed in a 4% (w/v) paraformaldehyde solution at room temperature (Alfa Aesar) for 15 min and permeabilized in 0.1% Triton X-100 in PBS for 2 min. The cells were then incubated with the relevant primary antibodies diluted in blocking solution (10% FBS in FCS) for 1 h at room temperature or at 4°C overnight. The cells were again washed in PBS and incubated with blocking solution for 5 min. They were then incubated with secondary antibodies for 1 h. After a final washing step, the coverslips were mounted on ProLong Gold (Molecular Probes) supplemented with the nuclear staining reagent DAPI overnight at 37°C. Detailed analysis of single cells was either performed by confocal microscopy (Zeiss LSM 780) and analyzed with IMAGEJ software or super-resolution 3D SIM imaging performed on an DeltaVision OMX V4 system (Applied Precision) equipped with an Olympus 60X numerical aperture (NA) 1.42 objective, cooled sCMOS camera and 405, 488 and 642 nm diode lasers. Z-stacks covering the whole cell were recorded with a Z-spacing of 125 nm. A total of 15 raw images (five phases, three rotations) per plane were collected and reconstructed by using SOFTWORX software (Applied Precision). For live cell imaging, cells were seeded in 50 mm MatTek glass bottom dishes. Images were captured under controlled CO_2_ conditions at 37°C with a DeltaVision microscope (Applied Precision), equipped with a live cell Elite TruLight Illumination System and cooled Photometrics CoolSNAP HQ2 CCD camera. Optical sections were acquired by using a 60X objective (Olympus, Plan Fluor, NA 1.42) in 0.6 μm steps on the z-axis on a motorized stage, and de-convolved by using SOFTWORX software (Applied Precision).

### Measurement of protein and DNA synthesis

HeLa cells were washed with leucine-free HEPES-buffered medium and incubated with increasing concentrations of PSL1a or PSL1a (C208A) mutant for 4 h at 37°C. Cells were then incubated with leucine-free HEPES-buffered medium complemented with 2 μCi/ml [^3^H]leucine (PerkinElmer) at 37°C for 20 min before proteins were precipitated with 5% (w/v) trichloroacetic acid and washed once with the same solution. Finally, the proteins were dissolved in 0.1 M KOH and radioactively labeled leucine incorporation was quantified by β-counting with a Tri-Carb 2100TR^®^ Liquid Scintillation Analyzer (Packard Bioscience). DNA synthesis was performed and measured essentially as described above, by using medium complemented with 2 μCi/ml [^3^H]thymidine.

### Immunoblotting

HeLa cells treated with PSL1a WT or mutant toxin were washed in ice cold PBS and lysed in lysis buffer (0.1 M NaCl, 10 mM Na_2_HPO_4_ pH 7.4, 1 mM EDTA, 1% Triton X-100) supplemented with a mixture of complete protease inhibitors (Roche Diagnostics). The nuclei were removed by centrifugation (14000 rpm, 5 min) and the lysates were boiled in Laemmli sample buffer. The proteins were separated by SDS-PAGE on a 4–20% gradient gel (Protean-TGX, Bio-Rad) under reducing conditions and blotted onto a PVDF membrane (Bio-Rad). The membranes were blocked by drying and then incubated overnight with primary antibodies diluted in 5% (w/v) BSA in TBS-T (tris buffered saline with 0.05% Tween 20) at 4°C. Thereafter, the membranes were washed with TBS-T and incubated for 45 min with HRP-conjugated secondary antibodies diluted in 5% non-fat dry milk followed by a final wash with TBS-T at room temperature. The following antibodies were used: LC3B (cat. no #2775), PARP (cat. no #9542), and caspase 3 (cat. no #9665) from Cell Signaling Technologies, and actin (cat. no #CLT9001, Cedarlane). The signals were detected by using the SuperSignal West Dura Extended Duration Substrate (Thermo Fisher) and a Syngene Chemi-Genious (Syngene).

### Statistical analysis

Student’s t-test or the Mann-Whitney rank sum test was used to calculate the *P*-value for all experiments. A *P*-value of ≤0.05 was considered to be statistically significant. A minimum of three independent experiments was performed and quantification of the data is given as mean +/- SEM. For quantification of cell rounding, between 90 and 300 cells per condition and time point have been analyzed in each independent experiment.

## Results

### PSL1a treatment leads to cell rounding in mammalian cells

**Fig 1:**
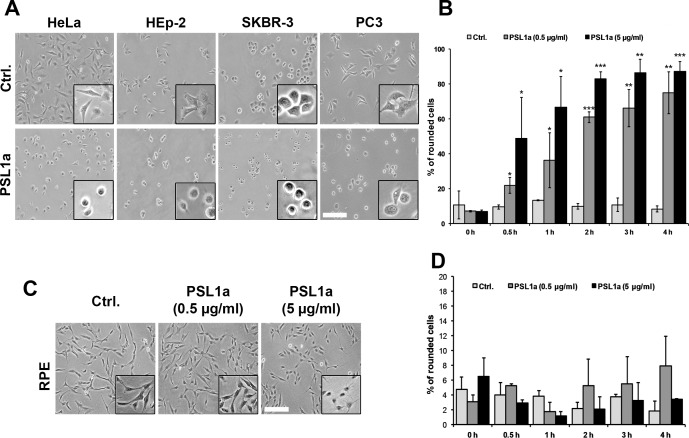
PSL1a treatment leads to cell rounding. (A) HeLa, HEp-2, SKBR-3 and PC3 cells were treated with 5 μg/ml of PSL1a in serum-free medium and incubated for 4 h at 37°C. PSL1a treatment leads to cell rounding in all tested cell lines. Scale bar: 200 μm. (B) HeLa cells were treated with 0.5 μg/ml and 5 μg/ml of PSL1a in serum-free medium at 37°C for 4 h in total. The cell morphology was analyzed by light microscopy at given time points and the numbers of rounded cells were quantified. Results are shown as mean values +/- SEM from at least three independent experiments; 180–300 cells per condition, per time point in each experiment (*P<0.05; **P<0.01; ***P<0.001 compared to untreated control). (C) RPE cells were treated for 4 h with 0.5 μg/ml and 5 μg/ml of PSL1a in serum-free medium at 37°C. Only slight morphological changes but no cell rounding could be observed even upon treatment with 5 μg/ml of PSL1a. Scale bar: 200 μm. (D) Quantification of rounded RPE cells after treatment with PSL1a, as described in (C). No significant cell rounding was observed. Results are shown as mean values +/- SEM from at least three independent experiments (210–300 cells per condition, per time point in each experiment).

To investigate the effects of PSL1a on cells, different cancer cell lines (HeLa, HEp-2, SKBR-3 and PC3) were treated with PSL1a at a concentration of 5 μg/ml and incubated for 4 h at 37°C. Cells were observed by light microscopy. After 4 h of PSL1a incubation, most cells were rounded up, but did not completely detach from the substratum. Cell rounding upon PSL1a treatment was observed in all cell lines tested (Fig 1A). Next, the time- and concentration-dependent effects of PSL1a were analyzed. We selected HeLa cells for these follow-up experiments, since they have a flat morphology, which is beneficial for quantitative microscopy analysis. HeLa cells were treated with two different concentrations of PSL1a (0.5 μg/ml and 5 μg/ml) and incubated for 4 h at 37°C, and the number of rounded cells was quantified at different time points by light microscopy. After 4 h of incubation 80–90% of the cells were rounded regardless of the PSL1a concentration (Fig 1B). However, at earlier time points cell rounding was more potently induced by the high PSL1a concentration. In order to test the effect of PSL1a also on non-cancer cells, we performed experiments with immortalized RPE (retinal pigment epithelium; hTERT RPE-1) cells. In contrast to all other tested cancer cells, we did not observe any strong morphological changes nor cell rounding in RPE cells (Fig 1C and 1D) after a 4 h treatment with PSL1a (0.5 and 5 μg/ml). However, long-term treatment with PSL1a resulted in cell rounding (24 h) and finally cell death (48 h) of RPE cells (S1 Fig), indicating a strongly reduced sensitivity of this non-cancer cell line.

These results demonstrate that PSL1a treatment leads to cell rounding and cell detachment in mammalian cancer cells in a time- and concentration-dependent manner. Since we did not observe any significant cell-type specific differences in the tested cancer cell lines upon PSL1a treatment, we focused on HeLa cells in all following experiments.

### PSL1a binding and cell rounding is inhibited in the presence of serum

As the cell-binding and toxic effect of lectins such as MOA and ricin is sensitive to the presence of serum [29, 30], we wanted to investigate the effect of serum also on the interaction of PSL1a with cells.

**Fig 2:**
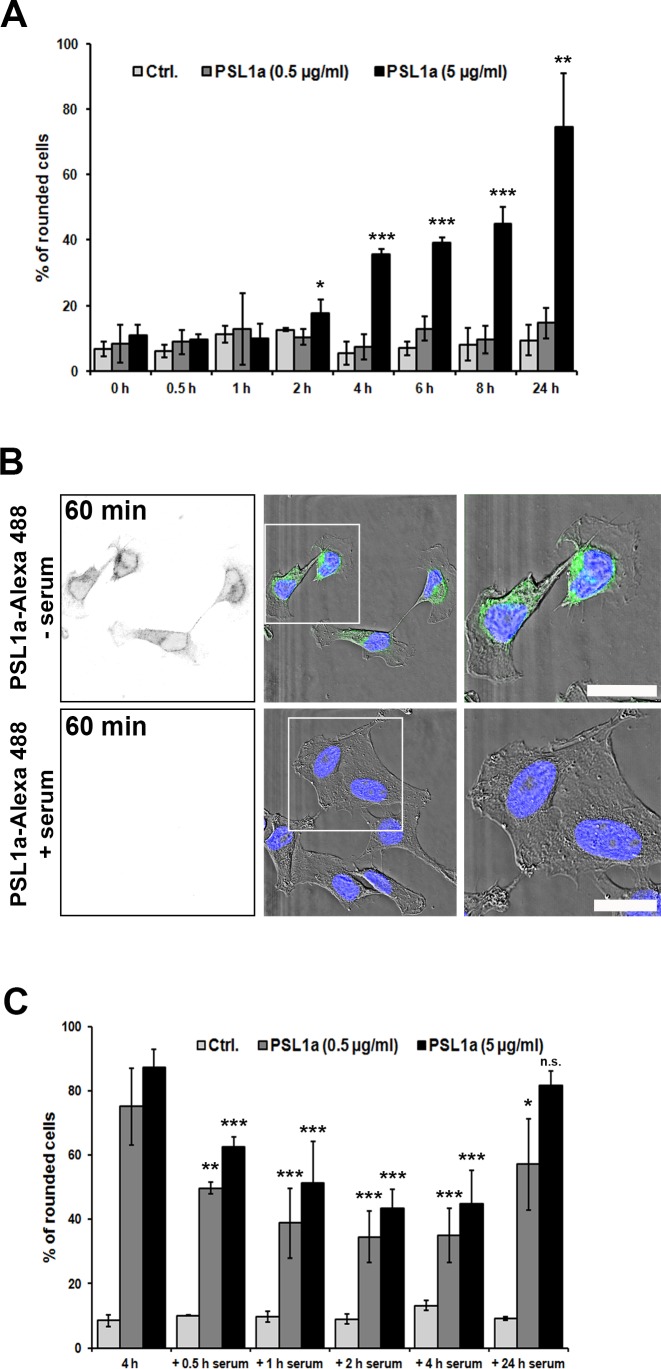
PSL1a induced cell rounding is partially inhibited by the presence of serum. (A) HeLa cells were treated with 0.5 μg/ml or 5 μg/ml of PSL1a in the presence of serum for 24 h in total. After the indicated time points the number of rounded cells was quantified. The presence of serum completely inhibits the effect of PSL1a at the lower concentration. At higher concentrations the effect of PSL1a is strongly reduced and the cellular response is delayed (compare Fig 1B and Fig 2A). Results are shown as mean values +/- SEM from at least three independent experiments (90–180 cells per condition, per time point in each experiment, *P<0.05; **P<0.01; ***P<0.001 compared to untreated control). (B) HeLa cells were treated with 1 μg/ml of Alexa-488 labeled PSL1a in the presence or absence of 10% serum for 60 min at 37°C. Subsequently, cells were washed, fixed and analyzed by fluorescence microscopy. After 60 min of incubation without serum PSL1a binds to cells (fluorescence signal in the left panel) and induces morphological changes (upper panel). In contrast, no binding of PSL1a is observed in the presence of serum (lower panel). Scale bars: 20 μm. (C) HeLa cells were incubated with 0.5 μg/ml or 5 μg/ml of PSL1a in serum-free medium. After 4 h of PSL1a treatment, the medium was supplemented with serum to a final concentration of 10% and the cells were incubated for additional 24 h. At the indicated time points the number of rounded cells was quantified. The cells gradually recovered from the rounded morphology within 2 h after addition of serum. After longer incubation (24 h) the protective effect of serum is abolished. The results are shown as mean values +/- SEM from at least three independent experiments (120–200 cells per condition, per time point in each experiment *P<0.05; **P<0.01; ***P<0.001 compared to cells treated for 4 h without serum).

To this end, HeLa cells were treated with PSL1a in the permanent presence of serum for up to 24 h (Fig 2A). After different time-points the number of rounded cells was quantified. Toxin-induced cell rounding was either completely inhibited at low PSL1a concentrations (0.5 μg/ml PSL1a) or strongly delayed at high concentrations (5 μg/ml). Thus, in the presence of serum the percentage of rounded cells after 24 h of treatment with high PSL1a concentrations (Fig 2A) was comparable to a 4 h treatment in serum-free conditions (Fig 1B).

As serum glycoproteins might directly bind PSL1a and thereby compete with binding of PSL1a to the cells, we wanted to see whether the inhibitory effect of serum on PSL1a-induced cell rounding is due to reduced toxin binding to the cells. For this purpose, cells were treated with 1 μg/ml fluorescently labeled PSL1a (PSL1a-Alexa488) for 60 min in the presence or absence of serum. Indeed, the presence of serum totally abolished the binding of PSL1a to the cell surface and no obvious morphological changes could be observed (Fig 2B).

Next, HeLa cells were incubated with PSL1a in serum-free medium for 4 h. Afterwards the medium was supplemented with fetal bovine serum (final concentration of 10% v/v) and the cells were incubated in the presence of toxin for additional 24 h. We observed that already 30 min after addition of serum the number of rounded cells was significantly reduced, independently of the PSL1a concentration used. 4 h after addition of serum, only 35–45% of the cells showed a rounded morphology (Fig 2C). By live-cell imaging we were able to demonstrate that upon addition of serum to the medium a fraction of formerly rounded cells were able to reattach to the substratum and recover their normal cellular morphology. Furthermore, some of the rounded cells were not only able to reattach, but also performed cell division (S1 File). However, the protective effect of serum disappeared after longer incubation times (24 h), and the number of rounded cells was increased (Fig 2C).

### PSL1a is internalized by mammalian cells and accumulates in intracellular vesicles

**Fig 3:**
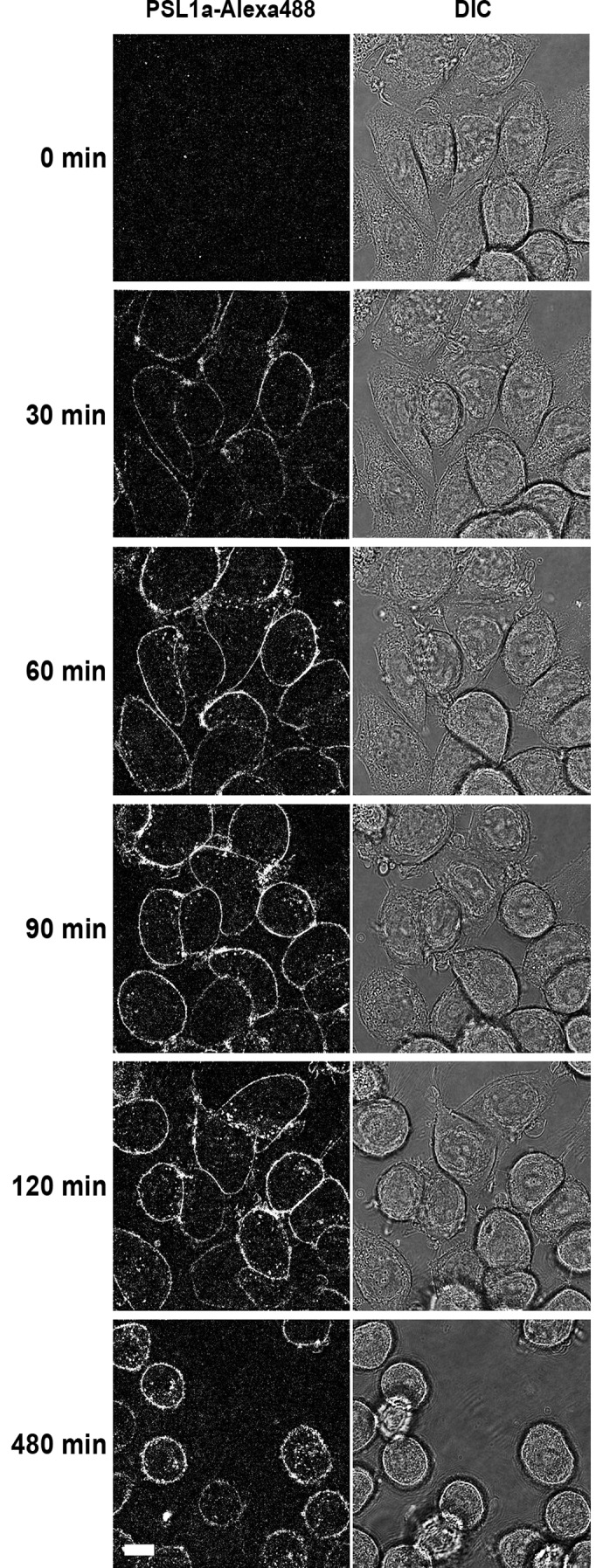
PSL1a is internalized and accumulates in intracellular vesicles. HeLa cells were treated with Alexa-488 labeled PSL1a (0.5 μg/ml) in serum-free medium and analyzed by live-cell imaging. PSL1a rapidly binds to the plasma membrane, and 60 min after addition PSL1a appears in intracellular vesicles and accumulates over time. Scale bar: 10 μm.

In a recent publication it was demonstrated that the fungal lectin MOA is internalized and accumulates in the perinuclear region [31]. Accordingly, we investigated whether PSL1a is also internalized by cells upon binding to the plasma membrane. For this purpose, HeLa cells were incubated with Alexa488-labeled PSL1a (0.5 μg/ml), and the toxin uptake was followed by live cell imaging. After 30 minutes, surface-bound toxin was clearly detectable (Fig 3). Approximately at the same time as morphological changes started to occur, PSL1a could be detected in intracellular vesicles, and then accumulated further with time.

### PSL1a does not disrupt the actin cytoskeleton or the microtubule network

**Fig 4:**
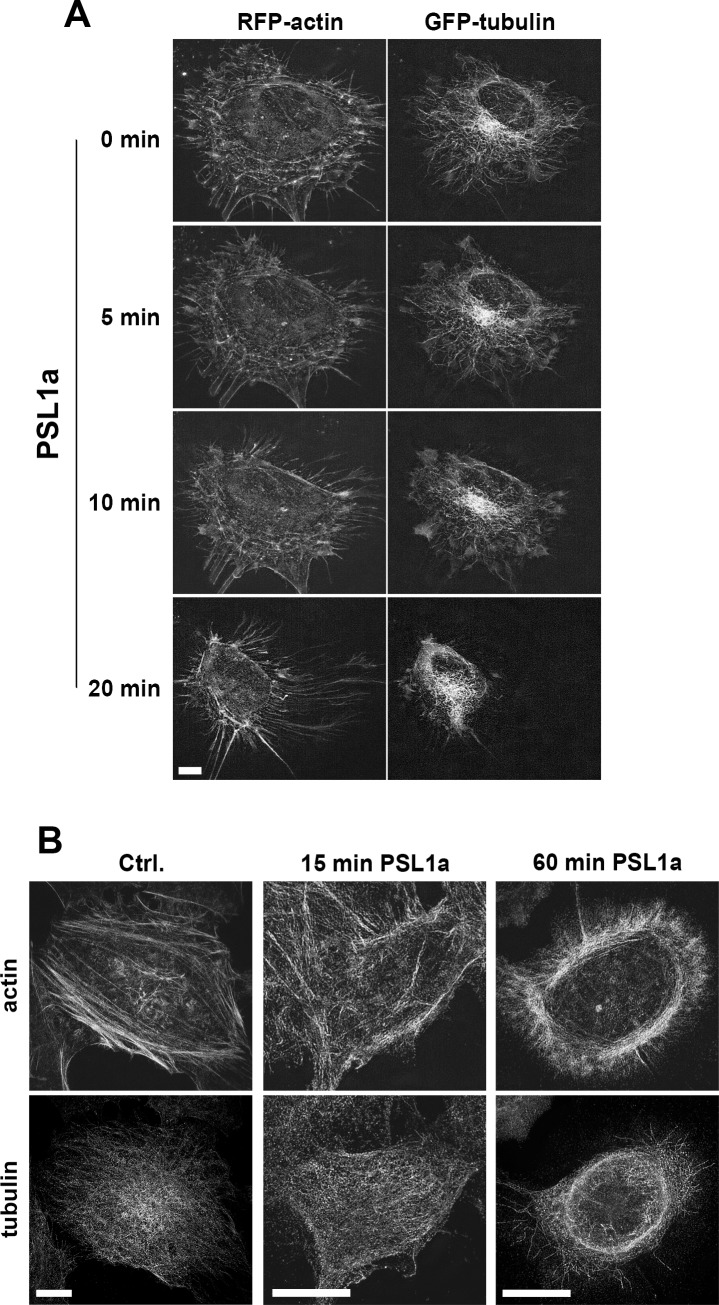
PSL1a treatment does not lead to disruption of actin filaments or the tubulin network. (A) HeLa cells were transiently transfected with mRFP-actin and β-tubulin-GFP. Fluorescence live cell imaging was performed to analyze the effects on filament dynamics after treatment with 0.5 μg/ml PSL1a in serum-free medium. PSL1a treatment leads to significant morphological changes, but does not induce disruption or fragmentation of actin or tubulin filaments. Scale bar: 7 μm. (B) HeLa cells were treated for 15 or 60 minutes with PSL1a, subsequently fixed and stained for F-actin and β-tubulin and analyzed by 3D SIM. PSL1a treatment induces cell rounding, but cells still contain a filamentous actin and tubulin network. Scale bars: 10 μm.

In order to probe the mechanisms by which PSL1a treatment leads to cell rounding, we investigated the effect of PSL1a on the actin cytoskeleton and on microtubules. Actin filaments and microtubules are important structural components of the cells, and interference with these components is associated with morphological changes. Several toxins have been described to directly target actin or microtubuli, for example, the C2 toxin of *Clostridium botulinum* is directly targeting actin, leading to the disruption of actin filaments and cell rounding [32]. The effect of PSL1a on actin and microtubuli was examined by live cell microscopy. To that end, HeLa cells were transiently transfected with RFP-actin and GFP-β-tubulin and treated with PSL1a or left untreated. Although cells treated with PSL1a rapidly changed from a flat and outspread to a more rounded morphology, these cells still contained an intact actin cytoskeleton and microtubule network with no obvious disruptions or fragmentations of these structural components (Fig 4A). Also super-resolution microscopy demonstrated that the actin cytoskeleton does not undergo disruption or fragmentation upon PSL1a treatment (Fig 4B). Thus our data indicate that the morphological changes induced by PSL1a are not consequences of a direct interference with the actin cytoskeleton or the microtubule network.

### PSL1a treatment leads to disruption of focal adhesion points

**Fig 5:**
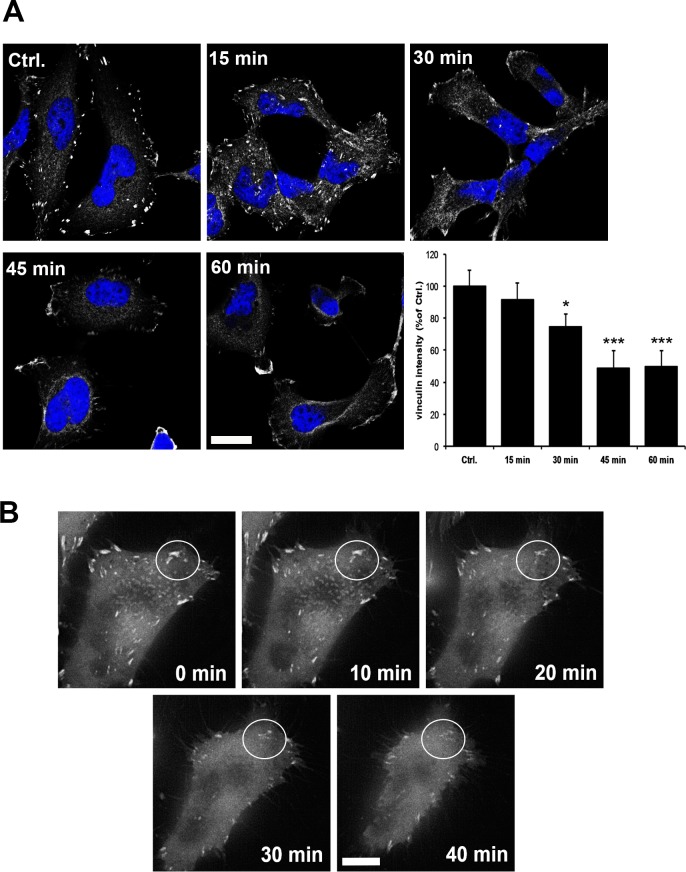
Vinculin positive focal adhesion points disappear upon treatment with PSL1a. (A) Confocal analysis of HeLa cells treated with 1 μg/ml PSL1a in serum-free medium for 30 min, fixed and stained for vinculin. PSL1a treatment leads to a strong reduction in the number and intensity of intracellular vinculin patches. Scale bar: 20μm. The quantification of vinculin intensity per cell upon treatment with PSL1a (1 μg/ml, 15–60 min) shows a significant reduction after 30 min. All data represent mean values +/- SEM from at least three independent experiments (90–120 cells per condition, per time point in each experiment, * *P*<0.05; *** *P*<0.001 compared to untreated control). (B) HeLa cells were transfected with RFP-vinculin and dynamic changes were analyzed by live cell imaging (see S1 File). PSL1a treatment leads to disappearance of vinculin patches (marked area) in a time-dependent manner. Scale bars: 10 μm.

To further investigate the cause of PSL1a-induced cell rounding, we analyzed the effect on focal adhesion points. Focal adhesion points are multiprotein complexes important for the transmission of mechanical forces by linking the actin cytoskeleton via the cellular membrane to the extracellular matrix [33]. Among others, the protein vinculin is one of the components associated with and localized to focal adhesions, making it a suitable marker to analyze the effect of PSL1a on these structures. For this purpose, HeLa cells were treated with 1 μg/ml PSL1a for 15–60 min, fixed and stained with specific antibodies for vinculin. In untreated cells RFP-vinculin was localized in discrete focal adhesion points, whereas upon PSL1a treatment, these vinculin patches disappeared in a time-dependent manner (Fig 5A). The quantification of the vinculin intensity per cell showed a significant reduction starting 30 min after toxin treatment (Fig 5A). In order to visualize the changes of vinculin upon PSL1a treatment in a dynamic way, we also performed live-cell fluorescence microscopy of RFP-vinculin expressing HeLa cells treated with 1 μg/ml of PSL1a. In line with the data from fixed cells, we observed a clear reduction of vinculin at focal adhesions after 20–30 min, accompanied by membrane retraction and cell rounding (Fig 5B and S2 File). These data indicate a PSL1a-induced disruption of focal adhesion points resulting in cell rounding.

### PSL1a inhibits both protein and DNA synthesis in mammalian cancer cells

**Fig 6:**
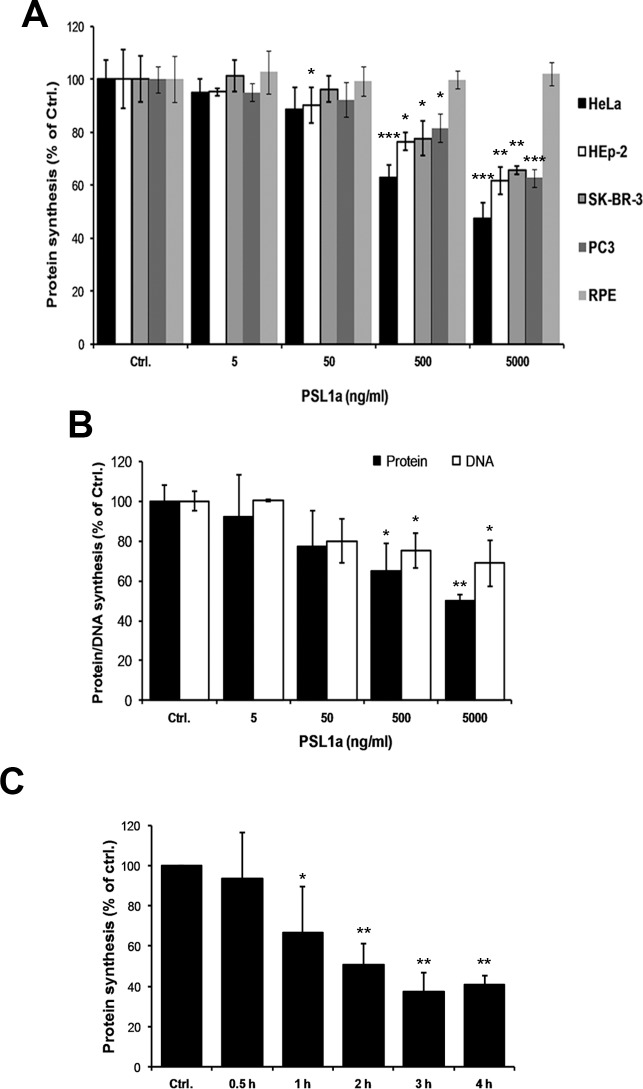
PSL1a treatment inhibits protein and DNA synthesis in mammalian cells. (A) HeLa, Hep-2, SKBR-3, PC3, and RPE cells were incubated with varying concentrations (5 ng/ml to 5 μg/ml) of PSL1a in serum-free medium for 4 h at 37°C. The level of protein synthesis was determined as described in the Experimental procedures. In contrast to all other tested cell lines, protein synthesis was not affected in non-cancer RPE cells. (B) HeLa cells were incubated with varying concentrations (5 ng/ml to 5 μg/ml) of PSL1a for 4 h at 37°C. The level of DNA synthesis, in parallel to the level of protein synthesis, was determined as described in Experimental procedures. All data represent mean values +/- SEM from at least three independent experiments (* *P*<0.05; ** *P*<0.01; *** *P*<0.001 compared to untreated control). (C) HeLa cells were treated with 5 μg/ml of PSL1a in serum-free medium at 37°C and the level of protein synthesis was measured at the indicated time-points. All data represent mean values +/- SEM from three independent experiments (* *P*<0.05; ** *P*<0.01 compared to untreated control).

To further investigate the toxic effects of PSL1a, we assessed both protein- and DNA synthesis. In all the mammalian cancer cell lines tested, a 4 h treatment with PSL1a (5 ng/ml to 5 μg/ml) reduced the protein synthesis 40–50% in a concentration-dependent manner compared to untreated cells (Fig 6A). A parallel evaluation of protein and DNA synthesis in PSL1a treated HeLa cells was also carried out (Fig 6B). Furthermore, protein synthesis upon PSL1a treatment (5 μg/ml) is reduced in a time-dependent manner in HeLa cells. The data suggest that PSL1a treatment reduces both protein and DNA synthesis to a similar extent. In line with a lack of significant cell rounding, even upon treatment with 5 μg/ml PSL1a for 4 h did not affect protein synthesis in non-cancer RPE cells. Thus, in our experiments PSL1a-induced cell rounding correlates with reduced protein and DNA synthesis. Together, these parameters can be used as a measure of PSL1a-induced cytotoxicity and might indicate a specificity of PSL1a towards mammalian cancer cells.

### Cytotoxicity of PSL1a is dependent on its proteolytic activity

**Fig 7:**
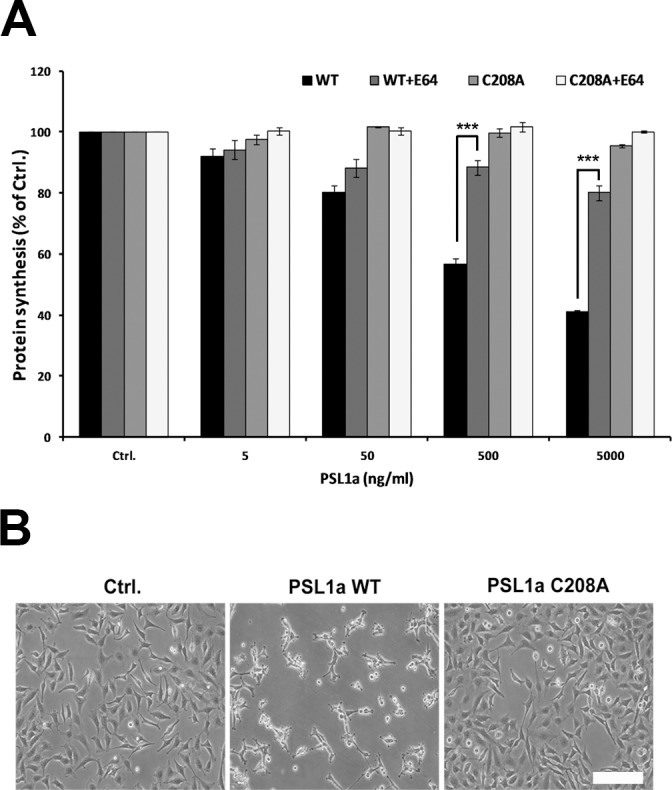
Impact on protein synthesis upon treatment with PSL1a (WT) and PSL1a (C208A) mutant. (A) HeLa cells were incubated with the indicated concentrations of PSL1a (WT) or PSL1a (C208A) mutant in the presence or absence of E-64 (10 μM) and incubated for 4 h at 37°C. The level of protein synthesis was determined as described in the Experimental procedures. The data represent mean values +/- SEM from at least three independent experiments (*** *P*< 0.001). (B) HeLa cells were incubated with 5 μg/ml of PSL1a (WT) or PSL1a (C208A) in serum-free medium at 37°C. Microscopic images were captured after 18 h of incubation. Treatment with PSL1a (WT) leads to morphological changes and a reduced cell number, whereas PSL1a (C208) mutant does not induce cytotoxic effects. Scale bar: 200 μm.

A structural alignment of PSL1a and MOA points to Cys208 as the nucleophile active in the proteolytic reaction. To probe whether the cysteine-protease activity of PSL1a plays a role in cellular toxicity, we compared the cytotoxicity of WT PSL1a with that of the PSL1a (C208A) variant, where the catalytic residue Cys208 is mutated to Ala208. In PSL1a (C208A) treated HeLa cells, the protein synthesis inhibitory effect was totally abolished (Fig 7A), and even after treatment for 18 h, cells appeared healthy with no sign of cell rounding (Fig 7B). In contrast, treatment with WT PSL1a strongly reduced the number of cells and affected the cell morphology of all remaining cells (Fig 7B).

To further confirm the role of the proteolytic activity in mediating cytotoxicity, we tested the effect of PSL1a and PSL1a (C208A) on protein synthesis in the presence or absence of the irreversible cysteine protease inhibitor E-64. Indeed, treatment with E-64 significantly protected against the effect of PSL1a WT toxin on protein synthesis (Fig 7A). Altogether, these results strongly indicate that the proteolytic activity of PSL1a is the main determinant of its cytotoxicity.

### PSL1a induces apoptosis, but does not induce autophagy

**Fig 8:**
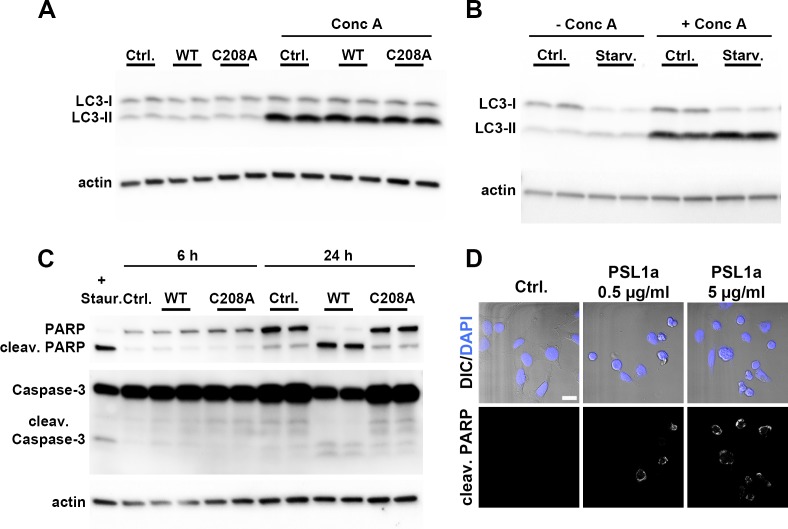
PSL1a induces apoptosis, but not autophagy. (A) HeLa cells were treated with 5 μg/ml of WT PSL1a or PSL1a (C208A) mutant in complete medium for 6 h in the presence or absence of 50 nM concanamycin A (Conc A). Lysates were prepared for immunoblotting and probed with the indicated antibodies. The blots shown are representative of three independent experiments. (B) HeLa cells were grown in full medium or starved in EBSS for 6 hours in the presence or absence of 50 nM Conc A. Lysates were prepared for immunoblotting and probed with the indicated antibodies. (C) HeLa cells were treated with 5 μg/ml of WT PSL1a or PSL1a (C208A) mutant in complete medium for 24 h at 37°C and the levels of PARP, caspase-3 and actin were detected by immunoblotting. The cleavage fragments of PARP and caspase-3 are indicated. Lysate of cells treated for 3 h with 1 μM staurosporine was used as a positive control (+ Staur.). (D) HeLa cells were treated with PSL1a at 0.5 or 5 μg/ml in serum-free medium. After 8 h incubation cells were fixed, stained for cleaved PARP by a specific antibody and analyzed by confocal microscopy. In contrast to untreated control cells, PSL1a treatment leads to cell rounding and induction of apoptosis as indicated by the appearance of cells positive for cleaved PARP. Scale Bar: 20 μm.

We wanted to see whether treatment with PSL1a would induce macroautophagy (here referred to as autophagy), a catabolic pathway often induced as a pro-survival mechanism to counteract stressful conditions, such as starvation [34]. Autophagy involves sequestration of cytoplasmic cargo into double-membrane vesicles termed autophagosomes, which fuse with lysosomes, leading to degradation of the contents by acidic hydrolases. Upon induction of autophagy, the cytosolic protein LC3-I is converted to the membrane-bound form LC3-II, which is regarded as an autophagy marker [35]. To investigate if PSL1a enhanced initiation of autophagy, we determined the transition from LC3-I to LC3-II by immunoblotting. As LC3-II is degraded in lysosomes after fusion between autophagosomes and lysosomes, the experiments were carried out also in the presence of the lysosomal inhibitor concanamycin A (Conc A) [35]. As expected, treatment with Conc A alone led to an accumulation of LC3-II, corresponding to the basal level of LC-II generation in these cells (Fig 8A and 8B). Moreover, starving the cells for amino acids, which is known to induce autophagy, increased the transition from LC3-I to LC3-II, and LC3-II accumulated when degradation was inhibited by Conc A (Fig 8B). In contrast, treatment with PSL1a did not alter the levels of LC3-I or -II, indicating that PSL1a treatment does not stimulate autophagy (Fig 8A). Similar data were obtained after 18 hours of PSL1a treatment (data not shown).

As cell rounding and loss of focal adhesion points might be associated with caspase activity and apoptosis induction, we wanted to know whether treatment with PSL1a induces apoptosis. To this end, the cleavage of PARP and caspase-3, as hallmarks of apoptosis, was detected by immunoblotting upon treatment with WT or mutant toxin. Although no cleavage of PARP or caspase-3 was detectable after 6 h of treatment with PSL1a WT, PARP was fully cleaved after 24 h of toxin exposure. Likewise, cleavage fragments of caspase-3 could be detected after 24 h upon WT PSL1a treatment (Fig 8C). Furthermore, at the 24 h time point a substantial fraction of the cells had detached from the plate. These cells were collected, and a pooled lysate consisting of both attached and detached cells was prepared. The catalytically inactive mutant C208A did not affect PARP at any time point. Cleavage of PARP was also observed upon treatment with 0.5 and 5 μg/ml WT PSL1a by confocal microscopy using a cleaved-PARP-specific antibody (Fig 8D).

Taken together, prolonged PSL1a treatment leads to potent apoptosis induction that is dependent on the cysteine protease activity of PSL1a.

## Discussion

The primary goal of our study was to investigate the cytotoxic potential of PSL1a and to examine the possible correlation between the proteolytic activity of PSL1a and its cytotoxicity in mammalian cells. A number of fungal lectins have been reported to exhibit cytotoxic activity in mammalian cells [12, 13, 15]. PSL1a and its structural homolog MOA [17] constitute two examples of cytotoxic lectins carrying a proteolytic domain. Recently, we described the cytotoxic effects of MOA on NIH/3T3 cells and found that MOA cytotoxicity is only partially dependent on its proteolytic activity [30]. Whereas, in this article we showed that for PSL1a proteolysis is of critical importance. In fact, we could demonstrate that PSL1a cytotoxicity is dependent on its catalytic cysteine residue (Cys208). Thus, even though both toxins exhibit a structurally conserved catalytic machinery (manuscript in preparation), they recognize different glycan structures [19, 36, 37] and exhibit their cytotoxic potential by slightly different mechanisms.

In contrast to the tested cancer cell lines, which were significantly affected by PSL1a treatment, no significant cell rounding or decrease in protein synthesis was observed in RPE non-cancer cells after 4–8 h of toxin treatment. These cells were not completely resistant to PSL1a treatment, but showed a strongly delayed response, as morphological changes could be observed only after a prolonged incubation with PSL1a (for 24 h). It has been reported that cancer cells often overexpress sialic acids on the cell surface [22]. Unfortunately, to our knowledge there are no comparative and quantitative studies describing the sialylation levels of the cell lines we used in our experiments. Thus, it has to be investigated further if the delayed toxicity and reduced sensitivity against PSL1a that we observed in non-cancer RPE cells are due to less sialic acids on the plasma membrane. Furthermore, it would be interesting to investigate to which extent other non-cancer cells are affected by PSL1a.

We observed that PSL1a-induced cytotoxicity is reduced in the presence of serum or could be reversed upon serum addition. Serum contains a rich variety of glycoproteins, growth factors and glucose, and our binding assay demonstrated that the presence of serum efficiently inhibits binding of PSL1a to the cell surface. It is possible that the serum glycoproteins directly bind to PSL1a, resulting in reduced toxic effects due to competition of PSL1a-binding to the cell surface. Alternatively, serum components might partially cover the cell surface receptors making them inaccessible to PSL1a, and thereby inducing cellular protection. It is also possible that protein addition may provide alternative targets for the proteolytic activity of PSL1a and in this way protect the cells.

Preliminary toxicity studies of PSL1a on model organisms suggest that PSL1a is toxic to *Drosophila melanogaster*, but non-toxic to *Caenorhabditis elegans* (our unpublished data). In *D*. *melanogaster* sialic acids are found on the outer cell surface, while *C*. *elegans* does not contain any sialic acid [38–40]. This might indicate that the presence of sialic acid on the cell surface determines PSL1a cytotoxicity. Further experiments are required to investigate this issue.

Besides binding of PSL1a to the cell surface, we also observed internalization and accumulation of PSL1a in intracellular vesicles by microscopic analysis. Whether the cytotoxic effects shown here require toxin internalization or whether surface-binding of the toxin is sufficient requires further studies. Importantly, it remains to be determined whether PSL1a has the ability to escape the endolysosomal pathway and be translocated to the cytosol in a manner similar to the plant lectin ricin [41], and thus have the ability to affect cytosolic targets. It is important to keep in mind that toxin molecules internalized into the endolysosomal pathway are still separated from the cell interior by the endosomal membrane.

Actin filaments and microtubules are important structural components that play a critical role in the regulation and maintenance of the cellular morphology. Several toxins have been reported to disrupt the actin cytoskeleton resulting in cell rounding [42–44]. However, we obtained no obvious disruption or fragmentation of the actin cytoskeleton or microtubule network in PSL1a-treated HeLa cells. This finding indicates that PSL1a has no direct effect on these structural networks. On the other hand, PSL1a treatment leads to the disruption of focal adhesions, as detected by the disappearance of vinculin patches. Focal adhesions are fundamental for cell adhesion and morphological integrity and anchor the cells to the substratum via integrins [45]. Thus, it is possible that cell rounding upon PSL1a treatment is a consequence of the disruption or disassembly of focal adhesions, associated with the loss of cellular attachment to the substratum. Interestingly, a recent publication demonstrated that MOA disrupts integrin-dependent cell adhesion in MDCKII cells followed by cell rounding [31]. Since MOA and PSL1a show structural and functional similarities, integrins might also be a possible substrate for PSL1a.

Our data show that besides the effects on the cellular morphology, PSL1a treatment also leads to reduced protein synthesis in a time- and concentration-dependent manner. It has been reported that decreased intracellular concentrations of ATP can lead to inhibition of protein synthesis [46]. However, in our preliminary experiments we did not observe any significant decrease of intracellular ATP levels after PSL1a treatment (5 ng/ml to 5 μg/ml, 4 h treatment), indicating that PSL1a does not induce ATP leakage or block ATP production. Thus, other and so far unknown mechanisms seem to be responsible for reduced protein synthesis upon PSL1a treatment. As integrins are also involved in signaling to the nucleus and act as translational regulators [47], the inhibition of protein synthesis by PSL1a could be an indirect effect of the disruption of integrin signaling. Furthermore, inhibition of protein synthesis is often accompanied by apoptosis, and we demonstrate that PSL1a induces apoptotic mechanisms. Interestingly, it has been reported that signal transduction regulated by integrin-mediated adhesion influences cell survival and prevents programmed cell death [48, 49]. Hence, it is possible that disruption of focal adhesions and interference with integrin signaling might indirectly inhibit protein biosynthesis and induce apoptosis.

In conclusion, our data demonstrate that PSL1a induces various cytotoxic responses in mammalian cells, such as reduced DNA- and protein synthesis, and eventually apoptosis. Importantly, PSL1a toxicity is almost entirely dependent on the proteolytic activity of the toxin.

## Supporting Information

S1 Fig Long-term incubation of RPE cells with PSL1a. RPE cells have been incubated with 0.5 and 5 μg/ml of PSL1a in serum-free medium for 24 and 48 h. The cell morphology was analyzed by light microscopy at given time points. In contrast to untreated control cells, PSL1a treatment leads to cell rounding after 24 h. After 48 h upon PSL1a treatment all cells were rounded up and partially detached from the substratum, indicating apoptotic cell death. Scale bar: 200 μm.
(TIF)

S1 File Time-lapse microscopy of recovering HeLa cells after PSL1a treatment. HeLa cells were incubated with 0.5 μg/ml PSL1a in serum-free medium. After 4 h of PSL1a incubation cells showed a rounded morphology, and the medium was supplemented with serum (time point 00:00) and identical cells were analyzed by live cell imaging with a DeltaVision microscope for additional 21 h (5 min/frame). PSL1a treatment in the absence of serum leads to cell rounding. After addition of serum, a fraction of the rounded cells is able to reattach and recover to a normal morphology. These recovered cells are motile and able to undergo cell division.
(AVI)

S2 File Time-lapse microscopy of RFP-vinculin upon PSL1a treatment. RFP-vinculin transfected HeLa cells were incubated with 1 μg/ml PSL1a in serum-free medium and visualized by live cell imaging with a DeltaVision microscope (30 s/frame). The RFP-vinculin intensity at focal adhesions decreased 20–30 min after treatment with PSL1a. This effect is accompanied by membrane retraction and initiation of cell rounding.
(AVI)
