# Supplementary figures and images for: Polyporus squamosus Lectin 1a (PSL1a) Exhibits Cytotoxicity in Mammalian Cells by Disruption of Focal Adhesions, Inhibition of Protein Synthesis and Induction of Apoptosis
Source: PLoS One. 2017 Jan 23;12(1):e0170716. doi: 10.1371/journal.pone.0170716 (PMC5256987; doi:10.1371/journal.pone.0170716)

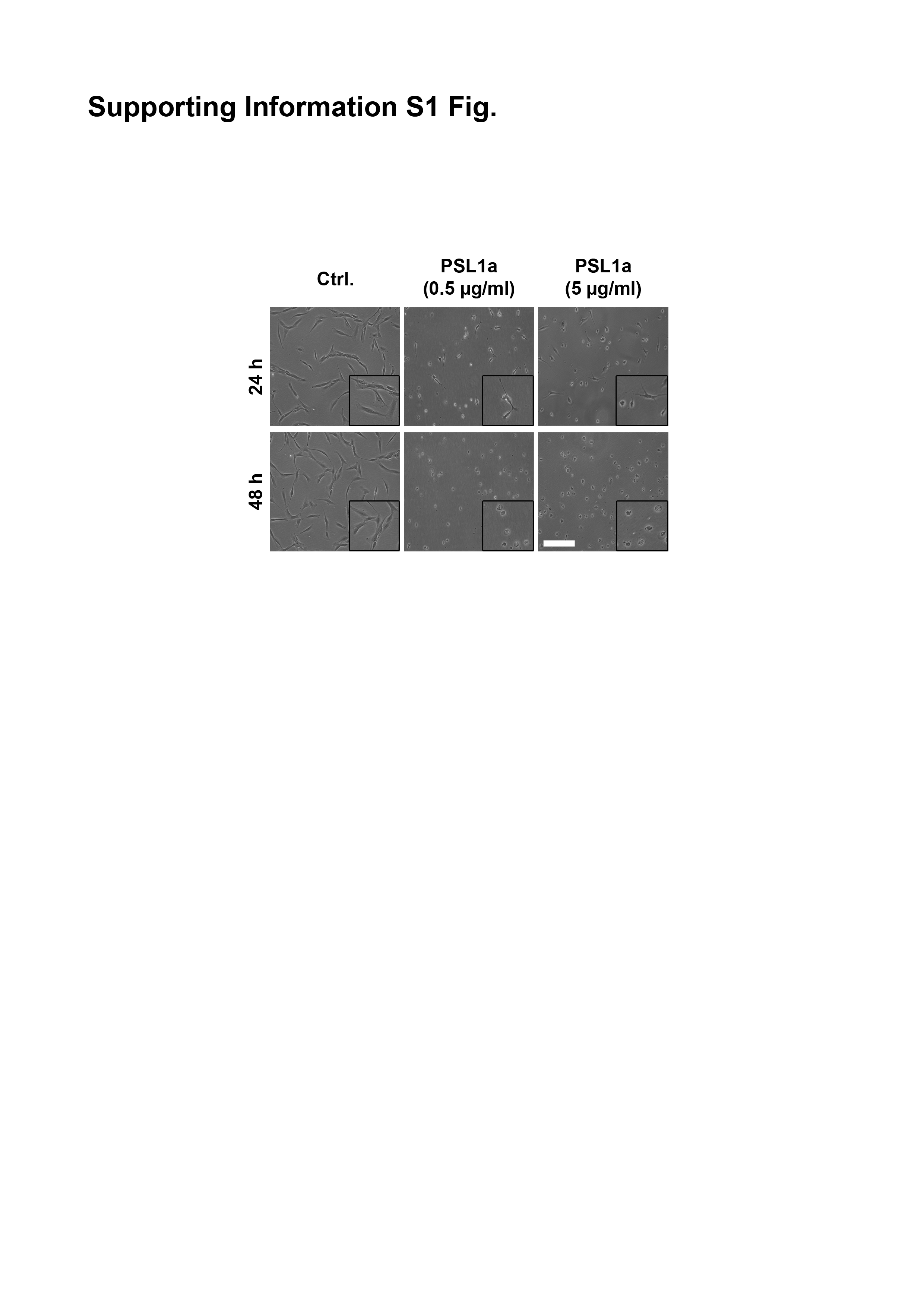

Supplement: S1 Fig — RPE cells have been incubated with 0.5 and 5 μg/ml of PSL1a in serum-free medium for 24 and 48 h. The cell morphology was analyzed by light microscopy at given time points. In contrast to untreated control cells, PSL1a treatment leads to cell rounding after 24 h. After 48 h upon PSL1a treatment all cells were rounded up and partially detached from the substratum, indicating apoptotic cell death. Scale bar: 200 μm. (TIF) [file pone.0170716.s001.tif]
